# HemaCisDB: An Interactive Database for Analyzing *Cis*-regulatory Elements Across Hematopoietic Malignancies

**DOI:** 10.1093/gpbjnl/qzae088

**Published:** 2024-12-26

**Authors:** Xinping Cai, Qianru Zhang, Bolin Liu, Lu Sun, Yuxuan Liu

**Affiliations:** State Key Laboratory of Experimental Hematology, National Clinical Research Center for Blood Diseases, Haihe Laboratory of Cell Ecosystem, Institute of Hematology & Blood Diseases Hospital, Chinese Academy of Medical Sciences & Peking Union Medical College, Tianjin 300020, China; Tianjin Institutes of Health Science, Tianjin 301600, China; State Key Laboratory of Experimental Hematology, National Clinical Research Center for Blood Diseases, Haihe Laboratory of Cell Ecosystem, Institute of Hematology & Blood Diseases Hospital, Chinese Academy of Medical Sciences & Peking Union Medical College, Tianjin 300020, China; Tianjin Institutes of Health Science, Tianjin 301600, China; State Key Laboratory of Experimental Hematology, National Clinical Research Center for Blood Diseases, Haihe Laboratory of Cell Ecosystem, Institute of Hematology & Blood Diseases Hospital, Chinese Academy of Medical Sciences & Peking Union Medical College, Tianjin 300020, China; Tianjin Institutes of Health Science, Tianjin 301600, China; State Key Laboratory of Experimental Hematology, National Clinical Research Center for Blood Diseases, Haihe Laboratory of Cell Ecosystem, Institute of Hematology & Blood Diseases Hospital, Chinese Academy of Medical Sciences & Peking Union Medical College, Tianjin 300020, China; Tianjin Institutes of Health Science, Tianjin 301600, China; State Key Laboratory of Experimental Hematology, National Clinical Research Center for Blood Diseases, Haihe Laboratory of Cell Ecosystem, Institute of Hematology & Blood Diseases Hospital, Chinese Academy of Medical Sciences & Peking Union Medical College, Tianjin 300020, China; Tianjin Institutes of Health Science, Tianjin 301600, China

**Keywords:** Database, *Cis*-regulatory element, Hematopoietic malignancy, Epigenetic marker, Transcriptional regulation

## Abstract

Non-coding *cis*-regulatory elements (CREs), such as transcriptional enhancers, are key regulators of gene expression programs. Accessible chromatin and H3K27ac are well-recognized markers for CREs associated with their biological function. Deregulation of CREs is commonly found in hematopoietic malignancies, yet the extent to which CRE dysfunction contributes to pathophysiology remains incompletely understood. Here, we developed HemaCisDB, an interactive, comprehensive, and centralized online resource for CRE characterization across hematopoietic malignancies, serving as a useful resource for investigating the pathological roles of CREs in blood disorders. Currently, we collected 922 assay of transposase accessible chromatin with sequencing (ATAC-seq), 190 DNase I hypersensitive site sequencing (DNase-seq), and 531 H3K27ac chromatin immunoprecipitation followed by sequencing (ChIP-seq) datasets from patient samples and cell lines across different myeloid and lymphoid neoplasms. HemaCisDB provides comprehensive quality control metrics to assess ATAC-seq, DNase-seq, and H3K27ac ChIP-seq data quality. The analytic modules in HemaCisDB include transcription factor (TF) footprinting inference, super-enhancer identification, and core transcriptional regulatory circuitry analysis. Moreover, HemaCisDB also enables the study of TF binding dynamics by comparing TF footprints across different disease types or conditions via web-based interactive analysis. Together, HemaCisDB provides an interactive platform for CRE characterization to facilitate mechanistic studies of transcriptional regulation in hematopoietic malignancies. HemaCisDB is available at https://hemacisdb.chinablood.com.cn/.

## Graphical abstract

**Graphical Abstract:**
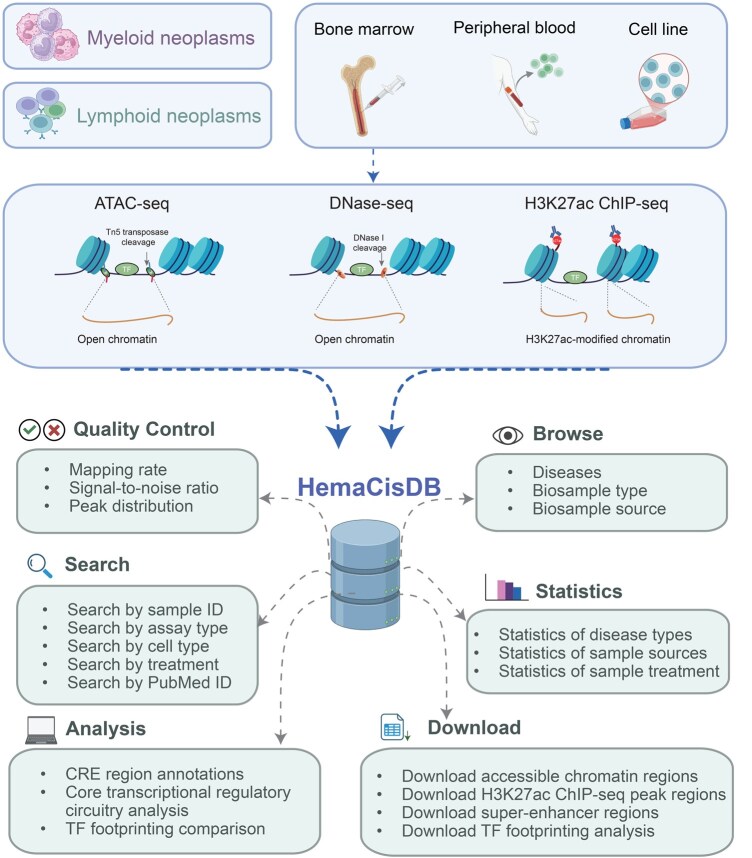

## Introduction

Mammalian gene expression programs require coordinated regulation of *cis*-regulatory elements (CREs), such as promoters, enhancers, silencers, and insulators. A promoter contains DNA sequences located immediately upstream and downstream of a gene to initiate gene transcription [1]. Enhancers are *cis*-regulatory DNA sequences which can loop over long genomic distances to interact with the promoters of target genes and activate gene expression in a distance-, orientation-, and position-independent manner [2]. Enhancers can be classified into three states, namely, inactive, poised, or active, based on their activities. An inactive enhancer is typically characterized by well-organized unmodified nucleosomes that obstruct the recruitment of transcription factors (TFs) and RNA polymerase. Poised enhancers, which are primed for activation, are often subject to mono-methylation of H3K4 (H3K4me1), which makes chromatin loose and more accessible. In contrast, fully activated enhancers, typically marked by H3K27 acetylation (H3K27ac) modification, exhibit fully open chromatin, facilitating the accessibility of RNA polymerase for bi-directional transcription initiation [3,4]. Silencers are non-coding genomic sequences which bind repressor proteins and prevent the transcription of target genes [5]. An insulator is a type of CRE that can mediate intra- and inter-chromosomal interactions [6]. CREs contain DNA motifs that are recognized and bound by sequence-specific DNA-binding proteins which in turn recruit co-factors to regulate target gene expression [1,7]. H3K27ac and chromatin accessibility detected by DNase I hypersensitive site sequencing (DNase-seq) or assay of transposase accessible chromatin with sequencing (ATAC-seq) are commonly used to identify candidate CREs, including promoters and active enhancers, which are the main focus of this study [7,8].

Emerging evidence shows that deregulation of CREs often contributes to the pathogenesis of hematopoietic malignancies. In T-cell acute lymphoblastic leukemia (T-ALL) patients, recurrent non-coding mutations that introduce *de novo* binding sites of MYB, a TF and oncoprotein, were found to initiate aberrant enhancer complexes and drive the expression of downstream oncogenes including *TAL1* and *LMO1* [9,10]. Another study with the analysis of 160 T-ALL cases identified recurrent focal duplications on chromosome 8q24 encompassing a *NOTCH1*-associated enhancer that regulates *MYC* expression [11]. In chronic lymphocytic leukemia (CLL) patients, a densely mutated intergenic region with enhancer characteristics was identified, and its disruption causes reduced expression of the B-cell-specific TF gene *PAX5* [12]. In acute myeloid leukemia (AML) patients with inv(3)/t(3;3), a *GATA2* enhancer rearrangement causes *GATA2* haploinsufficiency and *EVI1* activation simultaneously [13]. It was also found in leukemia cells that non-coding variants at *KRAS* and *PER2* enhancers affect the binding of nuclear receptor family TFs PPARG and RXRA, which disturbs the expression of *KRAS* and *PER2* and promotes the growth of leukemia cells [14]. Moreover, a recent study in diffuse large B cell lymphoma (DLBCL) showed that active super-enhancers (SEs) harboring hypermutation are linked to the upregulation of oncogenes related to B cell developmental and malignant transformation, including *BCL6*, *BCL2*, and *CXCR4* [15].

CRE landscape also serves as a useful biomarker to identify various subtypes of blood cancers and provides important information about potential cell-of-origin and/or targetable vulnerabilities [16–18]. Enhancer profiling in AML identified an active SE at the gene locus of *RARA* in 25% of samples, and its presence is associated with RARA-directed therapy responses [16]. Thus, the comprehensive and unbiased analysis of CREs is necessary to identify possible genetic and epigenetic causes of diseases, dissect regulatory mechanisms controlling disease initiation and progression, and stratify patients into subgroups for potential personalized medical care. Several databases have been developed to profile enhancer architecture and core transcriptional regulatory circuitry (CRC) in pan-cancer. These databases include Cistrome, ATACdb, dbSUPER, SEA, SEdb, and Cancer CRC [19–24]. Cistrome, a comprehensive database containing DNase-seq, ATAC-seq, and chromatin immunoprecipitation followed by sequencing (ChIP-seq) data for over 1500 TFs and histone marks, lacks disease-specific inquiries, making it challenging for users to locate datasets related to different types of hematopoietic malignancies [19]. Additionally, the Cistrome Cancer module, which is designed for enhancer/SE analyses, is only available for solid tumors. ATACdb, a repository of ATAC-seq data, profiles open chromatin regions across multiple cancer types, but has only a limited number of datasets related to hematopoietic malignancies [20]. The SEA, dbSUPER, and SEdb databases, curating SEs derived from H3K27ac ChIP-seq data across diverse cell types and tissues in various species, also contain a restricted number of datasets relevant to blood cancers [21–23]. Likewise, Cancer CRC, characterizing CRC through the integration of H3K27ac ChIP-seq and ATAC-seq data, is solely confined to solid tumors [24]. Therefore, a comprehensive, interactive, and centralized database that characterizes CREs in blood cancers is necessary but currently unavailable.

Here, we profiled CREs across myeloid and lymphoid neoplasms and developed a CRE database HemaCisDB (https://hemacisdb.chinablood.com.cn/) specific to hematopoietic malignancies. We collected 922 ATAC-seq, 190 DNase-seq, and 531 H3K27ac ChIP-seq datasets from cell lines and patient samples covering the full spectrum of leukemia and lymphoma subtypes, including Hodgkin’s lymphoma (HL), non-Hodgkin’s lymphoma (NHL), ALL, AML, CLL, chronic myeloid leukemia (CML), blastic plasmacytoid dendritic cell neoplasm (BPDCN), and multiple myeloma (MM). HemaCisDB is a user-friendly and interactive database allowing users to query, browse, and visualize *cis*-regulatory regions in datasets of interest. It provides comprehensive quality control (QC) metrics and analytical modules including TF footprinting, SE identification/ranking, and CRC analysis. HemaCisDB also allows users to compare TF footprints among user-defined datasets to explore TF binding dynamics across different disease types or conditions. By incorporating the latest epigenomic profiling datasets into HemaCisDB, we provide a comprehensive resource to facilitate the study of CREs and their functions in hematopoietic malignancies.

## Database construction

### Data collection

In HemaCisDB, we manually curated 1643 publicly available ATAC-seq (*n* = 922), DNase-seq (*n* = 190), and H3K27ac ChIP-seq (*n* = 531) datasets from blood cancer cell lines, patient samples with hematopoietic malignancies, and healthy donor samples. ATAC-seq/DNase-seq or H3K27ac ChIP-seq datasets were explored using the following keywords: “ATAC-seq” (or “DNase-seq” or “H3K27ac”), “*Homo sapiens*”, “AML” (or “acute myeloid leukemia”), “CML” (or “chronic myeloid leukemia”), “ALL” (or “acute lymphocytic leukemia”), “CLL” (or “chronic lymphocytic leukemia”), “MM” (or “multiple myeloma”), and “Lymphoma” against the Gene Expression Omnibus (GEO) database (http://www.ncbi.nlm.nih.gov/geo). All datasets were manually reviewed, and irrelevant data, such as single-cell ATAC-seq, were removed from the final list.

### ATAC-seq/DNase-seq and H3K27ac ChIP-seq data processing

Raw reads were trimmed using Trim Galore (v0.6.7) to remove low-quality sequences and adaptors. Trimmed reads were then mapped to human genome assembly (GRCh38, GENCODE release 39) using Bowtie 2 (v2.2.5) with default parameters [25]. For ATAC-seq/DNase-seq data, fragments mapped to mitochondrial DNA or with length greater than 2000 bp or less than 38 bp were filtered out by SAMtools (v1.6) [26]. Similarly, only fragments less than 2 kb in H3K27ac ChIP-seq data were kept for further analysis. Picard (v2.27.5) was applied to remove duplicated reads. Accessible chromatin regions and regions with H3K27ac modification were identified by MACS2 (v2.2.1.4) with parameters of “--nomodel --shift -100 --extsize 200” and default settings respectively [27]. ATAC-seq/DNase-seq and H3K27ac ChIP-seq peaks overlapping with the curated blacklisted regions (https://github.com/Boyle-Lab/Blacklist/tree/master/lists) were excluded from our database [28].

### ATAC-seq/DNase-seq QC

R packages ATACseqQC [29] and encodeChIPqc were used to evaluate ATAC-seq/DNase-seq data quality. Five QC metrics, including sequencing depth and library complexity assessment, size distribution of library insert fragments, enrichment score of transcription start site (TSS), nucleosome density distribution around TSS, and fraction of reads in peak (FRiP) were used in HemaCisDB. High-quality ATAC-seq data should (1) have a large proportion of insert fragments less than 100 bp which represent nucleosome-free regions, and the fragment size distribution should exhibit a clear periodicity corresponding to nucleosome-free regions and the occupancy of mono-nucleosomes, di-nucleosomes, and tri-nucleosomes; (2) show good TSS enrichment; (3) display strong enrichments of nucleosome-free fragments at TSS and nucleosome-bound fragments upstream and downstream of the active TSSs; and (4) show a high FRiP score with a high fraction of mapped reads falling within the peak regions.

### H3K27ac ChIP-seq QC

H3K27ac ChIP-seq data quality was assessed using R package ChIPQC [30]. FRiP, relative strand cross-correlation coefficient (RelCC), fragment length, and standardized standard deviation (SSD) were included in the QC metrics. FRiP, as calculated in ATAC-seq, represents mapped reads within called peaks, and high FRiP indicates high signal-to-noise ratio in the investigated data. RelCC is another “signal-to-noise” measurement which is determined from the calculation of strand cross-correlation, and RelCC > 1 suggests good signal-to-noise ratio. Fragment length estimated from ChIP-seq data should be consistent with fragment size selected in the library preparation step. SSD, calculated from the standard deviation of signal pile-up along chromosomes normalized to the total number of sequenced reads, represents the uniformity of coverage of reads along the genome, with a higher SSD indicative of better enrichment.

### Annotation and functional enrichment of H3K27ac ChIP-seq and ATAC-seq/DNase-seq peaks

R package ChIPseeker was used to annotate ChIP-seq and ATAC-seq/DNase-seq peaks [31]. The nearest genes of the peaks, the distances from the peaks to the TSSs of their nearest genes, and the genomic regions overlapping with the peaks are reported. The priorities of genomic annotation are as follows: promoter, 5′ untranslated region (UTR), 3′ UTR, exon, intron, downstream, and intergenic regions. Functional enrichment analysis was performed by R package clusterProfiler [32] based on the annotated nearest genes. Enriched pathways in biological processes of Gene Ontology (GO) and Kyoto Encyclopedia of Genes and Genomes (KEGG) were identified.

### TF footprinting analysis

In ATAC-seq/DNase-seq, DNA bases bound by TFs are protected from Tn5/DNase I cleavage within the binding sites, resulting in a relative depletion of signal within the open chromatin region, known as footprints. TF footprinting analysis allows for the prediction of precise TF binding sites. TOBIAS, which incorporates bias correction and footprinting, was used to perform TF footprinting analysis in HemaCisDB [33]. For each motif obtained from JASPAR, the number of binding sites found in peak regions and the mean footprint score for all TF binding sites are reported. A higher mean score indicates a clearer footprint of a TF. An aggregation plot across all sites for each TF was also generated for footprint visualization. Comparison of TF footprints across different samples and different conditions was also performed by TOBIAS, which allows users to investigate differential binding of TFs between different user-defined samples or conditions.

### SE identification

H3K27ac peaks were used to define SE boundaries, with the following filtering criteria: (1) exclusion of H3K27ac peaks that interact with ENCODE blacklisted genomic regions [28], and (2) exclusion of H3K27ac peaks located within ± 2 kb region of any RefSeq annotated gene promoter. SEs were then identified based on the H3K27ac ChIP-seq signals using the ROSE algorithm2 with the default parameters [34].

### CRC analysis

CRC analysis was performed using Python package coltron on samples with paired H3K27ac ChIP-seq and ATAC-seq data [35]. The command line to run coltron is as follows: coltron -e [output Enhancer file from ROSE] -b [H3K27ac ChIP-seq bam file] -g HG38 -n [Name] -s [Subpeak file] --promoter = True -o [Output]. And subpeaks in the aforementioned command are defined as ATAC-seq peaks that overlap with H3K27ac-marked enhancer regions.

Specifically, coltron applies the following strategy to identify autoregulatory and interconnected TF networks. The top 1000 enhancers ranked by H3K27ac signal were assigned to nearby genes whose promoters are highly acetylated. The underlying sequences of ATAC-seq peaks within H3K27ac enhancer regions were extracted and FIMO (v4.91) was employed to search TF node binding sites with position-weight matrices from TRANSFAC and JASPAR. An edge will be drawn between TF A and TF B if the motif of TF A is identified in an enhancer assigned to regulate TF B. In networks, the in-degree of TF A is defined as the number of TF nodes within any enhancers that regulate TF A. And the out-degree of TF A is described as the number of TF nodes regulated by an enhancer with the motif of TF A.

### SNP and eQTL annotation

Common single nucleotide polymorphisms (SNPs) and risk SNPs within accessible chromatin regions or enhancer regions were annotated. Common SNPs were obtained from dbSNP 156 [36], and SNPs with minimum allele frequency (MAF) < 0.01 were filtered out. Risk SNPs were retrieved from the GWAS Catalog [37], and risk SNPs associated with blood disorders were manually curated from all risk SNPs. Pairwise linkage disequilibrium (LD) was computed among SNPs with MAF > 0.05. PLINK (v2.0) [38] was employed to calculate the LD SNPs (*r*^2^ ≥ 0.8) in five super-populations, including African (AFR), Admixed American (AMR), East Asian (EAS), European (EUR), and South Asian (SAS). Expression quantitative trait loci (eQTLs) and eQTL–gene pairs were acquired from PancanQTL [39] and GTEx (v8.0) [40].

### Web portal

VueJS (v2.6.10, https://vuejs.org) and Element UI (v2.13.2, https://element.eleme.io/) were used to develop the front end of the server, and the back end was built in Python using the Django web framework (v3.2.8). The server was hosted on a Linux Ubuntu 20.04 server running Nginx/1.18.0. Intermediate results and pre-processed data files were stored on the local Ext4 file system, and MySQL (v8.0.31) database was used to store metadata and analysis results.

## Database content and usage

### Scheme of HemaCisDB

**Figure 1:**
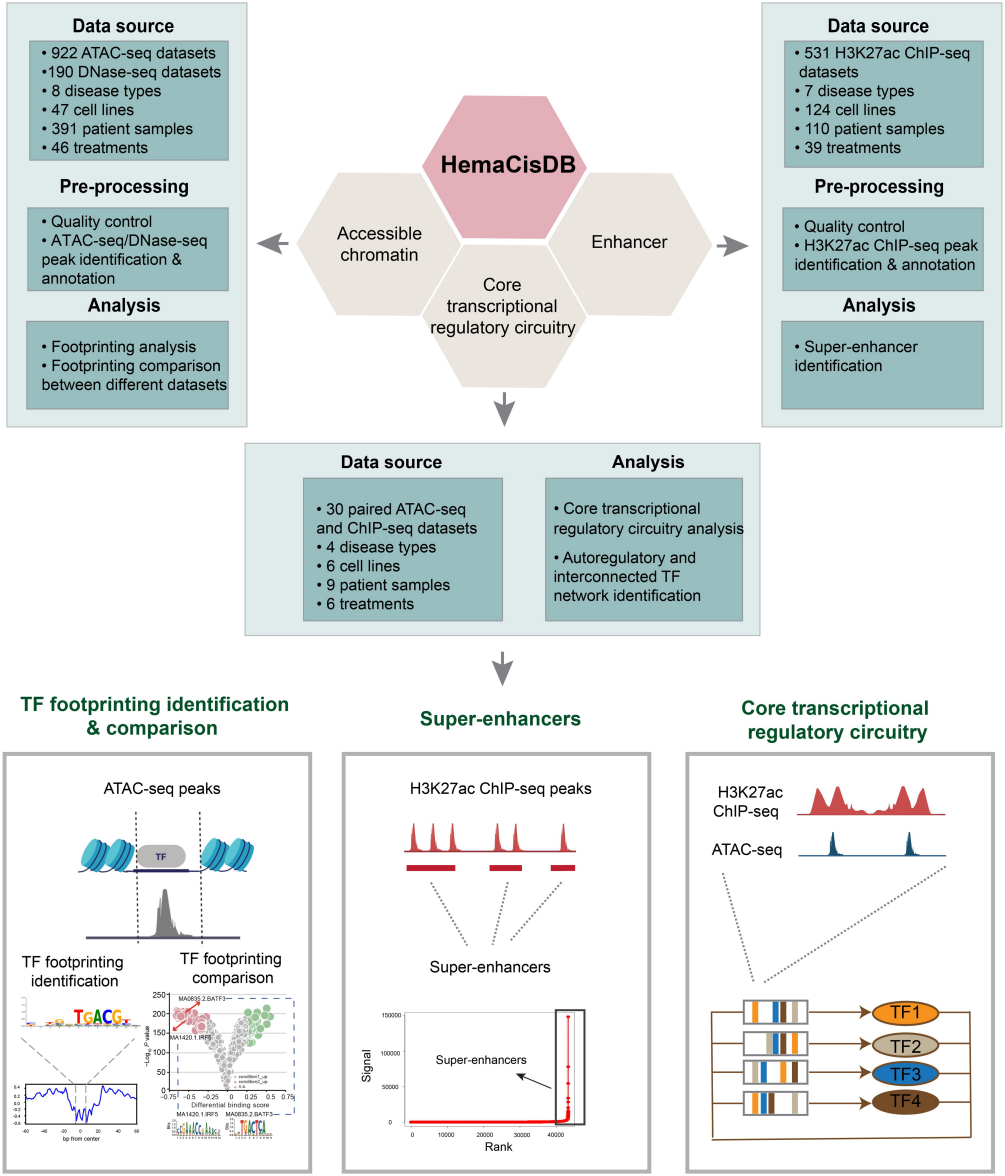
Scheme of HemaCisDB HemaCisDB contains three main modules: accessible chromatin, enhancer, and core transcriptional regulatory circuitry. ATAC-seq/DNase-seq data were employed to identify open chromatin regions, and H3K27ac ChIP-seq data were utilized for enhancer identification. The core transcriptional regulatory circuitry was identified from paired ATAC-seq and H3K27ac ChIP-seq data. Quality control, peak identification, and peak annotation were performed for each ATAC-seq/DNase-seq and H3K27ac ChIP-seq dataset. HemaCisDB provides analytical functions within these three modules. TF footprinting analysis and comparison are available in the “Accessible chromatin” module, super-enhancer identification is provided in the “Enhancer” module, and autoregulatory and interconnected TF networks can be identified in the “Core transcriptional regulatory circuitry” module. ATAC-seq, assay of transposase-accessible chromatin with sequencing; DNase-seq, DNase I hypersensitive site sequencing; H3K27ac, H3K27 acetylation; ChIP-seq, chromatin immunoprecipitation followed by sequencing; TF, transcription factor.

HemaCisDB currently contains 922 ATAC-seq, 190 DNase-seq, and 531 H3K27ac ChIP-seq datasets from 133 publications (**Figure 1**). We performed QC, peak identification, and peak annotation for each ATAC-seq, DNase-seq, and H3K27ac ChIP-seq dataset (Figure 1). As TF-bound DNA sequences are protected from Tn5-mediated transposition or DNase I digestion, leaving behind “footprints”, ATAC-seq and DNase-seq were proposed to be useful tools to infer genome-wide TF-bound locations [33,41]. HemaCisDB provides footprinting analysis for each ATAC-seq/DNase-seq dataset and allows users to explore TF binding information across diverse subtypes of hematopoietic malignancies (Figure 1).

SEs, which comprise clusters of enhancer elements, represent another important genomic feature that plays critical roles in driving cell-type-specific and disease-associated gene expression [42]. To uncover SEs that control gene programs related to different blood disorders, HemaCisDB enables SE identification by stitching closely distributed enhancers determined from H3K27ac ChIP-seq (Figure 1). For samples with paired ATAC-seq and H3K27ac ChIP-seq data, CRC analysis was performed to identify autoregulatory and interconnected TFs that regulate lineage-specific and disease-associated genes (Figure 1), thus providing information for elucidating malignant characteristics of different blood cancer subtypes [43,44].

### Datasets in HemaCisDB

**Figure 2:**
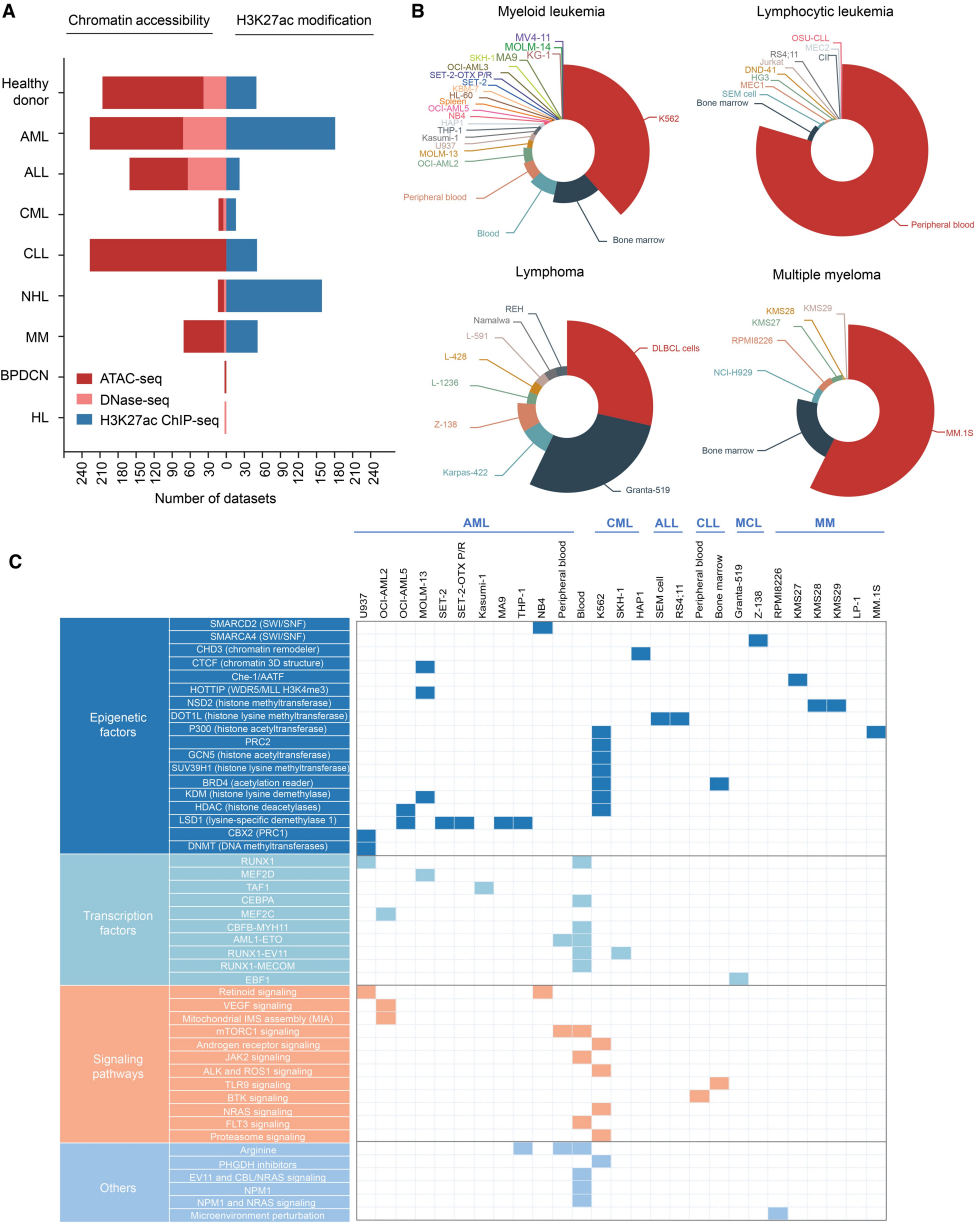
Statistics of datasets in HemaCisDB **A**. Number of ATAC-seq, DNase-seq, and H3K27ac ChIP-seq datasets summarized by disease type. **B**. Sample sources of ATAC-seq/DNase-seq datasets. **C**. Summarization of genetic or drug perturbations across different sample sources for ATAC-seq/DNase-seq. AML, acute myeloid leukemia; ALL, acute lymphocytic leukemia; CML, chronic myeloid leukemia; CLL, chronic lymphocytic leukemia; NHL, non-Hodgkin lymphoma; MM, multiple myeloma; BPDCN, blastic plasmacytoid dendritic cell neoplasm; HL, Hodgkin lymphoma; MCL, mantle cell lymphoma.

HemaCisDB contains ATAC-seq, DNase-seq, and H3K27ac ChIP-seq datasets from 294 healthy donor samples and 1349 disease-related samples (**Figure 2**A). Disease-related datasets include 8 subtypes of blood disorders across myeloid and lymphoid neoplasms, including AML, CML, ALL, CLL, MM, NHL, HL, and BPDCN (Figure 2A). NHL collected in the database consists of Burkitt’s lymphoma (BL), DLBCL, and mantle cell lymphoma (MCL). The datasets collected in HemaCisDB are from cell lines or patient samples. In detail, 43, 19, 53, and 14 cell lines were collected from diseases of myeloid leukemia, lymphocytic leukemia, lymphoma, and MM respectively (Figure 2B, Figure S1A). Patient samples are mainly derived from bone marrow or peripheral blood (Figure 2B). Treatment information of each dataset is also available in HemaCisDB, allowing users to investigate epigenetic landscape changes under different treatment conditions. Treatment categories mainly include genetic or pharmacological perturbations targeting epigenetic factors, TFs, and signaling pathways (Figure 2C, Figure S1B).

### A user-friendly interface for browsing, querying, and downloading datasets

**Figure 3:**
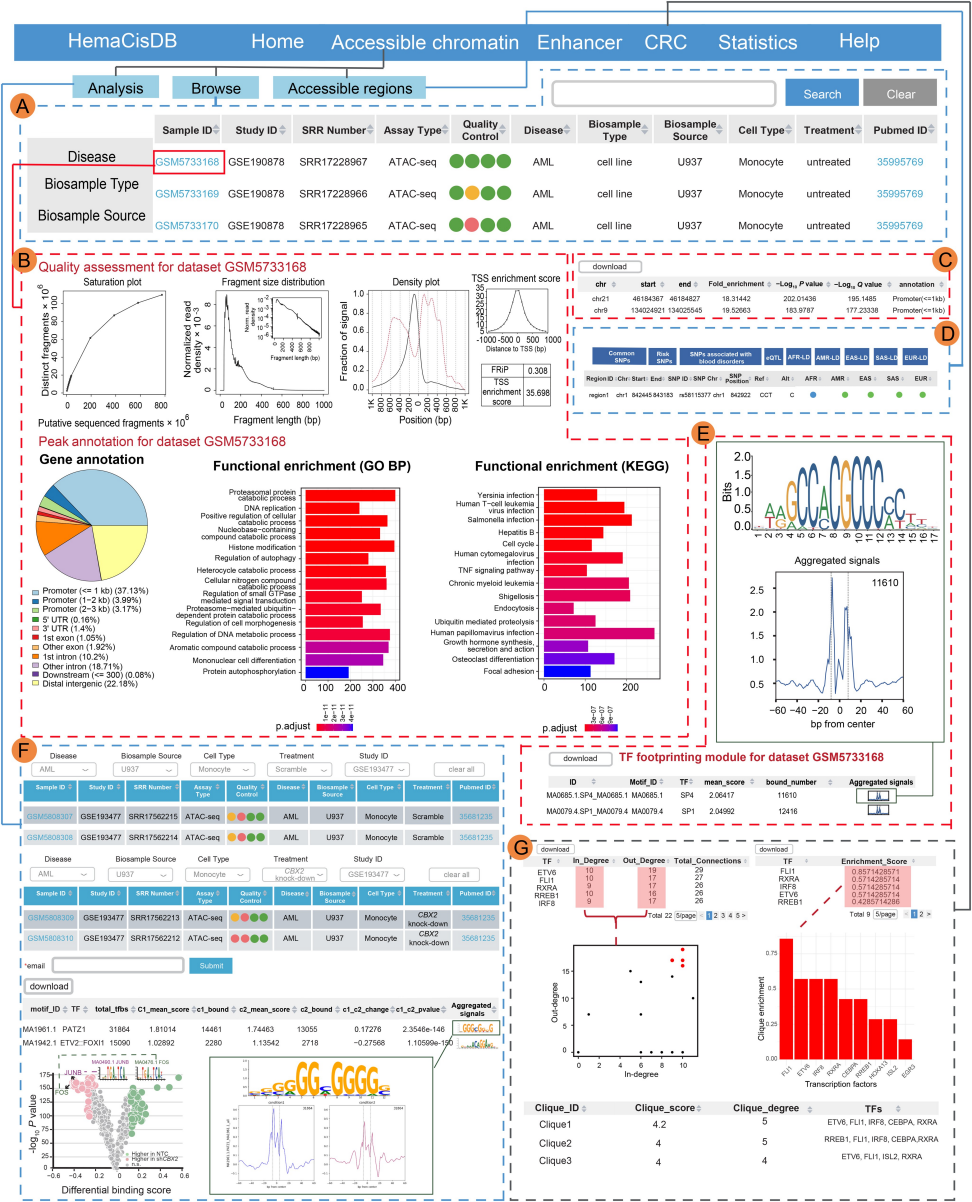
Data browsing and application modules for ATAC-seq/DNase-seq data **A**. Data browsing page for ATAC-seq/DNase-seq data. **B**. Quality assessment and functional annotation of peaks for selected ATAC-seq/DNase-seq dataset. **C**. ATAC-seq/DNase-seq peaks identified from selected dataset. Coordinates, fold enrichment, *P* value, *Q* value, and annotation of each peak are reported. **D**. Common SNPs, risk SNPs, and risk SNPs associated with blood disorders that overlap each accessible region are reported. For each common SNP, corresponding eQTL and LD SNPs in five super-populations (AFR, AMR, EAS, EUR, and SAS) are also reported. **E**. TF footprint inference page. The information of motif ID, TF name, mean footprint score across all TF binding sites, and the number of predicted binding sites in peak regions is reported. The aggregation plot and motif logo for each TF can be visualized. **F**. TF footprint comparison page. Disease type, biosample source, cell type, treatment, and study ID can be used to filter and select datasets to be compared. The link to comparison results will be sent to users via email. Differential binding of each TF between different samples or different conditions is reported. Each point in the scatter plot represents a TF, with x axis as the binding differences of a TF between two different user-selected samples and y axis as −log_10_  *P* value. **G**. CRC analysis page. The information of TF in-degrees, TF out-degrees, TF enrichment scores, and top-scoring TF cliques is reported. Each point in the scatter plot represents a TF, with x axis as the in-degree of a TF and y axis as the out-degree of a TF. Bar plot shows TFs ranked by clique enrichment scores. SNP, single nucleotide polymorphism; eQTL, expression quantitative trait loci; LD, linkage disequilibrium; AFR, African; AMR, Admixed American; EAS, East Asian; EUR, European; SAS, South Asian.

HemaCisDB allows users to browse samples and filter datasets by disease type, biosample type, and biosample source (**Figure 3**A, Figure S2A). A search option is also supported, allowing users to query a dataset through corresponding study ID, sample ID, disease type, cell line name, or other related information (Figure 3A, Figure S2A). Users can visualize detailed information for each ATAC-seq/DNase-seq or H3K27ac ChIP-seq dataset, including QC metrics, peak coordinates, and peak annotations (Figure 3B, Figure S2B). All peak regions and peak information are available for download from HemaCisDB (Figure 3C, Figure S2C). Furthermore, peak regions derived from ATAC-seq/DNase-seq or H3K27ac ChIP-seq across different datasets have been integrated and annotated. Detailed information for each region is accessible through the “Accessible regions” or “Enhancer regions” tabs under “Accessible chromatin” or “Enhancer”, respectively. Users can explore comprehensive annotations for each region, including common SNPs, risk SNPs, SNPs associated with blood disorders, eQTLs, and LD SNPs, by clicking on the “Region ID” (Figure 3D, Figure S2D). In addition, each region is annotated to indicate whether it is shared among multiple disease types or exclusive to a specific disease type across hematopoietic malignancies.

### Application modules

Four analytic modules were developed in HemaCisDB.

#### TF footprinting module

HemaCisDB provides TF footprint inference within accessible chromatin regions (Figure 3E). For each TF, the number of predicted binding sites in peak regions and mean footprint score across all TF binding sites are reported. Users can visualize footprints and motif logos for each TF via aggregation plots and sequence logo plots.

#### TF footprinting comparison module

HemaCisDB enables the comparative analysis of TF footprints between different samples (Figure 3F). Users can choose from the drop-down menu of disease types, cell types, or treatment conditions to select samples for comparisons. The output containing differentially bound TFs between different samples will be generated, and the link to the comparison results will be sent to users by email. Differences and *P* values for each TF will be reported, and users can visualize the summarized results through a volcano plot. For each TF, HemaCisDB also provides aggregation plots across different regions between different samples.

#### SE module

This module shows detailed SE information identified from H3K27ac ChIP-seq datasets (Figure S2E). The coordinates, constituent size, signal rank, and annotation of each SE are reported. Additionally, for each dataset, HemaCisDB generates a plot of stitched enhancers ranked by H3K27ac ChIP-seq signals, enabling users to visualize the distribution of SEs.

#### CRC analysis module

HemaCisDB provides CRC analysis to identify autoregulatory and interconnected TF networks for each sample with paired H3K27ac ChIP-seq and ATAC-seq data (Figure 3G). This module shows TF degrees, TF networks, clique enrichment of TFs, and detailed information of cliques with top clique scores. The degree for a given SE-regulated TF is defined as the sum of in-degree and out-degree of the TF. In-degree is calculated as the number of SE-regulated TFs that bind to the assigned SEs of the given TF. And out-degree is described as the number of TF-associated SEs that are bound by the given SE-regulated TF. A clique in TF networks is defined as a subnetwork containing at least four TF nodes that are connected to themselves and all other TFs within the network. “Clique enrichment” of a given TF is defined as the percentage of cliques with that TF, and “clique score” is calculated as the average of clique enrichment. In-degree and out-degree of each TF are reported and summarized by scatter plots (Figure 3G). Clique enrichment of TFs is listed and TFs with top clique enrichment scores are shown in ordered bar plots (Figure 3G). Users can visualize detailed information of top scoring cliques, including clique scores, clique degrees, and all TFs in that clique.

### CRE landscape across different subtypes of hematopoietic malignancies

**Figure 4:**
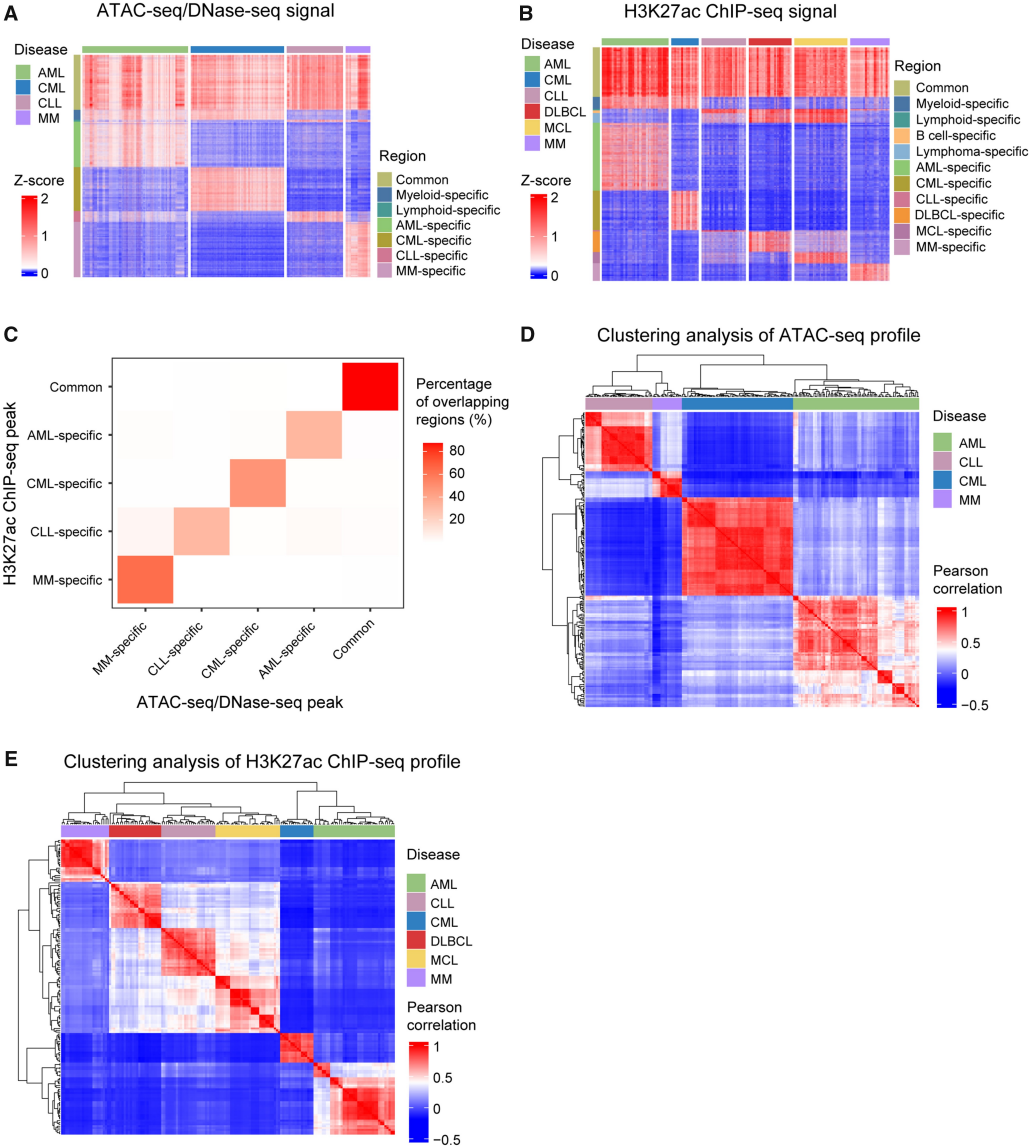
ATAC-seq/DNase-seq and H3K27ac ChIP-seq profiles across different types of hematopoietic malignancies **A**. ATAC-seq/DNase-seq signals within common and disease-specific peaks across different types of blood cancers. **B**. H3K27ac ChIP-seq signals within common and disease-specific peaks across different types of blood cancers. **C**. Overlaps between common/disease-specific regions derived from H3K27ac ChIP-seq peaks and ATAC-seq/DNase-seq peaks. The color scale represents the percentage of regions derived from H3K27ac ChIP-seq peaks that overlap with regions derived from ATAC-seq/DNase-seq peaks. **D**. Clustering analysis of ATAC-seq profiles across different cell lines, cell types, or disease lineages. **E**. Clustering analysis of H3K27ac ChIP-seq profiles across different cell lines, cell types, or disease lineages. DLBCL, diffuse large B cell lymphoma.

Integrative analyses were conducted to categorize ATAC-seq/DNase-seq and H3K27ac ChIP-seq peak regions across all compiled datasets into common regions shared by multiple disease types and disease-specific regions within the hematopoietic malignancies under comparison (**Figure 4**A and B). Each category of regions derived from H3K27ac ChIP-seq peaks exhibited the highest overlap with its corresponding counterpart derived from ATAC-seq/DNase-seq peaks (Figure 4C). Hierarchical clustering of the ATAC-seq/DNase-seq peak regions and H3K27ac ChIP-seq peak regions showed that cell lines and primary samples from myeloid neoplasms were grouped together, and samples derived from lymphoid malignancies exhibited higher similarities (Figure 4D and E). Thus, CRE landscape can successfully capture the information of lineages and disease types.

### Demonstration of HemaCisDB usage

**Figure 5:**
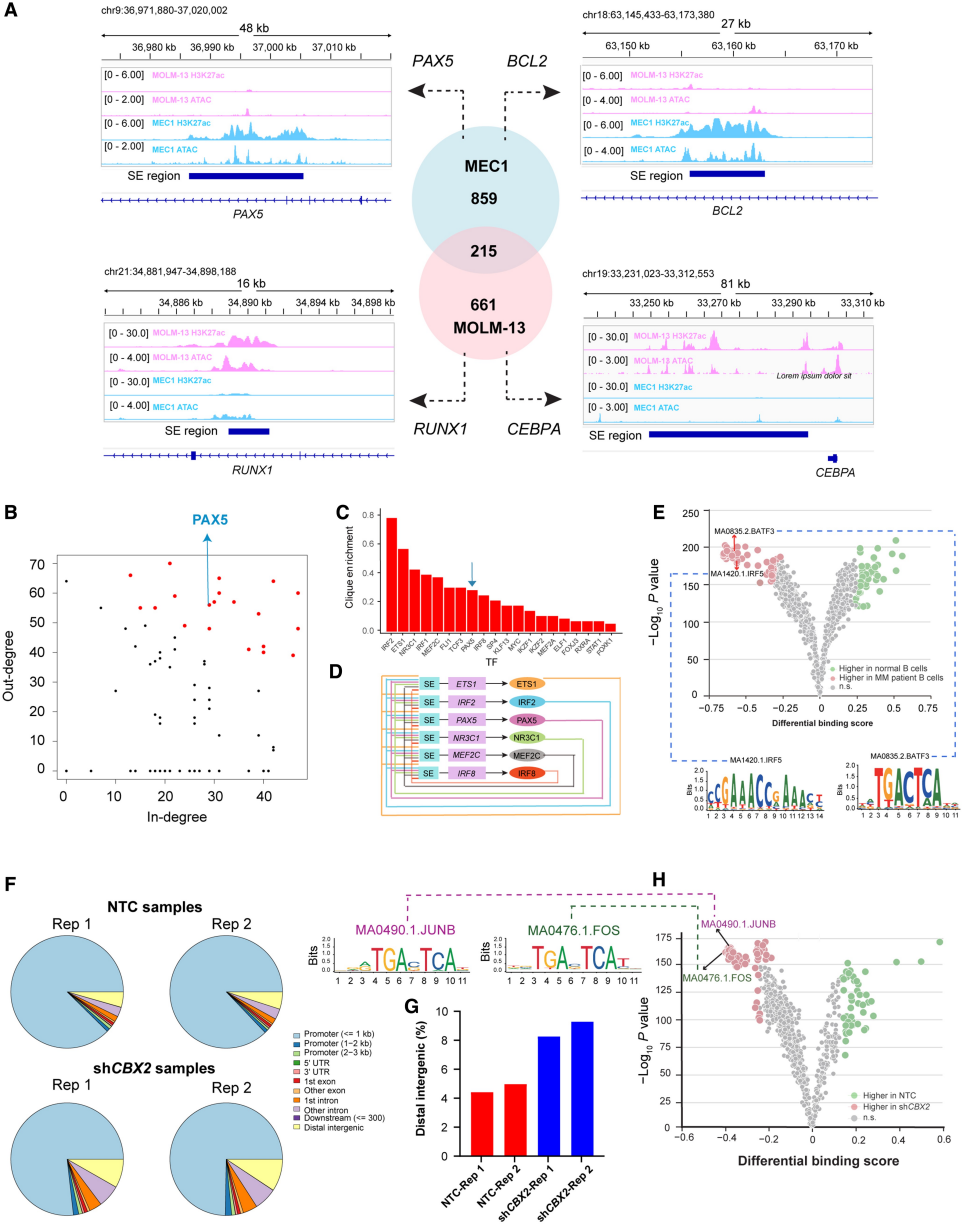
Demonstration of HemaCisDB usage by case studies **A**. SE identification in CLL cell line MEC1 and AML cell line MOLM-13. SEs were identified using the analytic module of “Super-enhancer Identification” in HemaCisDB. **B**. TF degree analysis performed by the “CRC analysis” module of HemaCisDB highlights PAX5 as a critical TF in CLL cell line MEC1 with relatively high TF in-degree and TF out-degree. Each point in the scatter plot represents a TF. **C**. TF enrichment score calculated by the “CRC analysis” module of HemaCisDB highlights PAX5 as an important TF in CLL cell line MEC1 with a relatively high TF enrichment score. Bar plot shows TFs ranked by clique enrichment scores. **D**. PAX5 is involved in top scoring TF clique in CLL cell line MEC1. TF cliques were identified from the “CRC analysis” module of HemaCisDB. **E**. Analysis performed by the “TF footprinting comparison” module of HemaCisDB shows significant enrichments of IRF5 and BATF3 motifs within open chromatin regions in MM tumor cells compared to normal B cells. Each point in the scatter plot represents a TF motif collected from JASPAR. **F**. Annotations of ATAC-seq peaks for AML cell lines transduced with sh*CBX2* or non-targeting control. NTC denotes the non-targeting control, and peak annotation for each ATAC-seq dataset is available at HemaCisDB. **G**. Percentage of ATAC-seq peaks located at the distal intergenic regions in AML cell lines transduced with sh*CBX2* or non-targeting control. **H**. Analysis performed by the “TF footprinting comparison” module of HemaCisDB shows significant enrichments of motifs associated with TFs JUNB and FOS at open chromatin sites in cell lines with *CBX2* silencing. Each point in the scatter plot represents a TF motif collected from JASPAR. SE, super-enhancer; Rep, repeat.

CRE studies in cancer can reveal critical transcriptional regulators and uncover the underlying molecular mechanisms driving cancer pathogenesis. Here, we showcase how the HemaCisDB database can be utilized to achieve these aims. Previous studies showed that CREs linked to lineage-specific genes are commonly dysregulated in hematopoietic malignancies [9,15]. HemaCisDB is a useful resource that facilitates the identification of cell-type-specific CREs and the prediction of prospective regulators in user-defined diseases. CLL is a type of adult leukemia that originates primarily from malignant B cells. Analysis using HemaCisDB revealed that SEs associated with B-cell-specific TF genes *PAX5* and *BCL2* are exclusively present in CLL compared to AML, as evidenced by enriched H3K27ac ChIP-seq and ATAC-seq signals in CLL cell line MEC1 and depleted signals in AML cell line MOLM-13 (**Figure 5**A). Conversely, SEs associated with TF genes involved in myeloid development, such as *RUNX1* and *CEBPA*, exhibited enriched H3K27ac ChIP-seq and ATAC-seq signals solely in AML (Figure 5A). Furthermore, CRC analysis in MEC1 highlighted PAX5 as a crucial transcriptional regulator with high TF degrees, high clique enrichment score, and involvement in top scoring autoregulatory TF networks (Figure 5B–D), signifying its potential roles in CLL pathogenesis. These findings align with a previous study indicating that PAX5 serves as a core regulator in CLL regulatory networks and plays an indispensable role in the maintenance of CLL cell viability [45].

Comparing malignant cells to their normal counterparts from healthy donors can offer valuable insights into the molecular mechanisms underlying cancer pathogenesis. HemaCisDB has curated ATAC/DNase-seq and H3K27ac ChIP-seq data from various purified cell types of healthy donors. Additionally, the TF footprinting comparison module in HemaCisDB allows for the comparison of TF footprints across different samples. We demonstrated the potential of HemaCisDB in identifying TF candidates that drive cancer pathogenesis by comparing TF footprints using ATAC-seq data of fluorescence-activated cell sorting (FACS)-purified myeloma cells from MM patients and normal B cells from primary human blood cells. This analysis revealed a significant enrichment of the IRF5 motif within open chromatin regions in MM cells (Figure 5E), consistent with previous findings that identified IRF5 regulon as a cancer-specific regulon with higher activity in MM cells *vs.* normal B cells using single-cell/single-nucleus RNA sequencing (RNA-seq) and single-nucleus ATAC-seq [46]. Additionally, the BATF3 motif was also found to be enriched within open chromatin regions in MM patient tumor cells (Figure 5E). The patient sample used for comparison harbors the t(11;14) translocation, aligning with prior findings that *BATF3* expression co-occurs with t(11;14) translocation [47]. *BATF3* expression has also been reported to correlate with worse progression-free survival (PFS), overall survival, and increasing clinical stages in MM [47]. These imply that IRF5 and BATF3, identified through HemaCisDB footprinting comparison, could potentially be critical TFs driving MM pathogenesis.

The employment of genetic perturbation methods, such as CRISPR knockout and short hairpin RNAs (shRNAs), has facilitated gene function investigations. HemaCisDB has also curated treatment information from collected datasets, including genetic or drug perturbations of genes that encode epigenetic factors, TFs, or proteins in signaling pathways. This provides a valuable resource to study how the dysfunction of different genes contributes to the pathogenesis of hematopoietic malignancies. Here, we demonstrate the utility of HemaCisDB in investigating how gene perturbations contribute to disease development through CRE deregulations. CBX family of proteins are canonical PRC1 subunits, which can read and bind to H3K27me3 modifications [48]. This, in turn, facilitates the recruitment of canonical PRC1 to PRC2 target genes, thereby leading to chromatin condensation and transcriptional repression. *CBX2* overexpression has been implicated in promoting the proliferation of cancer cells [49]. HemaCisDB revealed that *CBX2* silencing leads to an increase in open chromatin regions at distal intergenic regions in the AML cell line U937 (Figure 5F and G). Furthermore, the footprinting comparison of AML cell lines transduced with sh*CBX2* and non-targeting control revealed that motifs for AP-1 TFs, such as JUNB and FOS, were significantly enriched in open chromatin sites in cell lines with *CBX2* silencing (Figure 5H). AP-1 TFs, which act as effectors of receptor tyrosine kinase signaling and growth factor, are activated by the Ras/MAPK pathway [50], suggesting that CBX2 might promote AML through MAPK-associated regulatory sites. These results are consistent with a previous study that demonstrated that silencing *CBX2* results in epigenetic reprogramming of regulatory sites associated with p38 MAPK, leading to gene expression deregulation [51].

Collectively, HemaCisDB offers an interactive platform for CRE characterization, facilitating mechanistic investigations of transcriptional regulation in hematopoietic malignancies.

## Discussion

CRE landscape can serve as a useful biomarker to identify blood cancer subtypes. Dysregulation of CREs often contributes to pathogenesis and drug resistance of hematopoietic malignancies [52]. The comprehensive and unbiased characterization of CREs facilitates the analysis of putative cell-of-origin and/or targetable vulnerabilities of blood disorders. Databases, such as Cistrome, ATACdb, dbSUPER, SEA, SEdb, and Cancer CRC, have been developed to characterize CREs across various cell types and tissues in different types of cancers. However, these resources were mainly designed for pan-cancer analysis and only encompass a limited number of datasets relevant to hematopoietic malignancies (Table S1). Thus, we developed HemaCisDB, a comprehensive and interactive data portal with specific focus on CREs in hematopoietic malignancies. HemaCisDB supports disease-specific queries in blood cancers. In contrast to many previously published databases that solely collected ATAC-seq/DNase-seq or H3K27ac ChIP-seq data, HemaCisDB provides the most extensive collection of datasets and CRE profiles related to blood cancers (Table S1). This is achieved by integrating both ATAC-seq/DNase-seq and H3K27ac data, enabling concurrent characterization of open chromatin regions, enhancers/SEs, and CRCs (Table S1). HemaCisDB provides comprehensive QC metrics, facilitating the QC assessment for each dataset. Furthermore, HemaCisDB profiled treatment conditions, allowing investigations into how different treatments affect transcriptional regulation in blood disorders (Table S1). Additionally, HemaCisDB provides detailed annotations for each open chromatin and enhancer region, including genomic features, SNPs, eQTLs, and an indication of whether a region is shared among multiple disease types or exclusive to a specific disease type across hematopoietic malignancies (Table S1). These comprehensive annotations allow for in-depth functional exploration of each region. Application modules implemented in HemaCisDB allow users to compare different datasets to identify cell-type-specific CREs, predict potential regulators contributing to specific diseases, or identify regulators potentially affected by drug or genetic perturbations. Additionally, the CREs curated in HemaCisDB can also be integrated with other datasets, such as whole-genome sequencing (WGS) or RNA-seq data, to identify potential enhancer hijacking events or enhancer RNAs (eRNAs) that may act as prognostic biomarkers or therapeutic targets in hematopoietic malignancies. Taken together, HemaCisDB serves as a resource facilitating the hematology community in characterizing CREs and their roles in disease pathophysiology.

## Supplementary Material

qzae088_Supplementary_Data

## Data Availability

HemaCisDB is accessible at https://hemacisdb.chinablood.com.cn/. It has also been submitted to Database Commons [53] at the National Genomics Data Center (NGDC), China National Center for Bioinformation (CNCB), which is publicly accessible at https://ngdc.cncb.ac.cn/databasecommons/database/id/9953.

## CRediT author statement

**Xinping Cai:** Methodology, Software, Investigation, Data curation, Writing – original draft, Writing – review & editing. **Qianru Zhang:** Investigation, Data curation. **Bolin Liu:** Data curation. **Lu Sun:** Data curation. **Yuxuan Liu:** Conceptualization, Methodology, Writing – original draft, Writing – review & editing, Supervision, Project administration, Funding acquisition. All authors have read and approved the final manuscript.

## References

[1] Haberle V , Stark A. Eukaryotic core promoters and the functional basis of transcription initiation. Nat Rev Mol Cell Biol 2018;19:621–37. 29946135 10.1038/s41580-018-0028-8 PMC6205604

[2] Bulger M , Groudine M. Functional and mechanistic diversity of distal transcription enhancers. Cell 2011;144:327–39. 21295696 10.1016/j.cell.2011.01.024 PMC3742076

[3] Heinz S , Romanoski CE, Benner C, Glass CK. The selection and function of cell type-specific enhancers. Nat Rev Mol Cell Biol 2015;16:144–54. 25650801 10.1038/nrm3949 PMC4517609

[4] Chen H , Li C, Peng X, Zhou Z, Weinstein JN, Cancer Genome Atlas Research Network, et al A pan-cancer analysis of enhancer expression in nearly 9000 patient samples. Cell 2018;173:386–99. 29625054 10.1016/j.cell.2018.03.027 PMC5890960

[5] Pang B , van Weerd JH, Hamoen FL, Snyder MP. Identification of non-coding silencer elements and their regulation of gene expression. Nat Rev Mol Cell Biol 2023:24:383−95. 36344659 10.1038/s41580-022-00549-9

[6] Yang J , Corces VG. Chromatin insulators: a role in nuclear organization and gene expression. Adv Cancer Res 2011;110:43–76. 21704228 10.1016/B978-0-12-386469-7.00003-7 PMC3175007

[7] Spitz F , Furlong EE. Transcription factors: from enhancer binding to developmental control. Nat Rev Genet 2012;13:613–26. 22868264 10.1038/nrg3207

[8] Andersson R , Gebhard C, Miguel-Escalada I, Hoof I, Bornholdt J, Boyd M, et al An atlas of active enhancers across human cell types and tissues. Nature 2014;507:455–61. 24670763 10.1038/nature12787 PMC5215096

[9] Mansour MR , Abraham BJ, Anders L, Berezovskaya A, Gutierrez A, Durbin AD, et al An oncogenic super-enhancer formed through somatic mutation of a noncoding intergenic element. Science 2014;346:1373–7. 25394790 10.1126/science.1259037 PMC4720521

[10] Li Z , Abraham BJ, Berezovskaya A, Farah N, Liu Y, Leon T, et al APOBEC signature mutation generates an oncogenic enhancer that drives *LMO1* expression in T-ALL. Leukemia 2017;31:2057–64. 28260788 10.1038/leu.2017.75 PMC5629363

[11] Herranz D , Ambesi-Impiombato A, Palomero T, Schnell SA, Belver L, Wendorff AA, et al A NOTCH1-driven *MYC* enhancer promotes T cell development, transformation and acute lymphoblastic leukemia. Nat Med 2014;20:1130–7. 25194570 10.1038/nm.3665 PMC4192073

[12] Puente XS , Bea S, Valdes-Mas R, Villamor N, Gutierrez-Abril J, Martin-Subero JI, et al Non-coding recurrent mutations in chronic lymphocytic leukaemia. Nature 2015;526:519–24. 26200345 10.1038/nature14666

[13] Groschel S , Sanders MA, Hoogenboezem R, de Wit E, Bouwman BAM, Erpelinck C, et al A single oncogenic enhancer rearrangement causes concomitant *EVI1* and *GATA2* deregulation in leukemia. Cell 2014;157:369–81. 24703711 10.1016/j.cell.2014.02.019

[14] Li K , Zhang Y, Liu X, Liu Y, Gu Z, Cao H, et al Noncoding variants connect enhancer dysregulation with nuclear receptor signaling in hematopoietic malignancies. Cancer Discov 2020;10:724–45. 32188707 10.1158/2159-8290.CD-19-1128 PMC7196497

[15] Bal E , Kumar R, Hadigol M, Holmes AB, Hilton LK, Loh JW, et al Super-enhancer hypermutation alters oncogene expression in B cell lymphoma. Nature 2022;607:808–15. 35794478 10.1038/s41586-022-04906-8 PMC9583699

[16] McKeown MR , Corces MR, Eaton ML, Fiore C, Lee E, Lopez JT, et al Superenhancer analysis defines novel epigenomic subtypes of non-APL AML, including an RARα dependency targetable by SY-1425, a potent and selective RARα agonist. Cancer Discov 2017;7:1136–53. 28729405 10.1158/2159-8290.CD-17-0399 PMC5962349

[17] Wong RWJ , Ngoc PCT, Leong WZ, Yam AWY, Zhang T, Asamitsu K, et al Enhancer profiling identifies critical cancer genes and characterizes cell identity in adult T-cell leukemia. Blood 2017;130:2326–38. 28978570 10.1182/blood-2017-06-792184 PMC5701524

[18] Bhagwat AS , Lu B, Vakoc CR. Enhancer dysfunction in leukemia. Blood 2018;131:1795–804. 29439951 10.1182/blood-2017-11-737379 PMC5909760

[19] Taing L , Dandawate A, L’Yi S, Gehlenborg N, Brown M, Meyer CA. Cistrome Data Browser: integrated search, analysis and visualization of chromatin data. Nucleic Acids Res 2024;52:D61–6. 37971305 10.1093/nar/gkad1069 PMC10767960

[20] Wang F , Bai X, Wang Y, Jiang Y, Ai B, Zhang Y, et al ATACdb: a comprehensive human chromatin accessibility database. Nucleic Acids Res 2021;49:D55–64. 33125076 10.1093/nar/gkaa943 PMC7779059

[21] Khan A , Zhang X. dbSUPER: a database of super-enhancers in mouse and human genome. Nucleic Acids Res 2016;44:D164–71. 26438538 10.1093/nar/gkv1002 PMC4702767

[22] Chen C , Zhou D, Gu Y, Wang C, Zhang M, Lin X, et al SEA version 3.0: a comprehensive extension and update of the Super-Enhancer archive. Nucleic Acids Res 2020;48:D198–203. 31667506 10.1093/nar/gkz1028 PMC7145603

[23] Wang Y , Song C, Zhao J, Zhang Y, Zhao X, Feng C, et al SEdb 2.0: a comprehensive super-enhancer database of human and mouse. Nucleic Acids Res 2023;51:D280–90. 36318264 10.1093/nar/gkac968 PMC9825585

[24] Wei L , Chen J, Song C, Zhang Y, Zhang Y, Xu M, et al Cancer CRC: a comprehensive cancer core transcriptional regulatory circuit resource and analysis platform. Front Oncol 2021;11:761700. 34712617 10.3389/fonc.2021.761700 PMC8546348

[25] Langmead B , Salzberg SL. Fast gapped-read alignment with Bowtie 2. Nat Methods 2012;9:357–9. 22388286 10.1038/nmeth.1923 PMC3322381

[26] Li H , Handsaker B, Wysoker A, Fennell T, Ruan J, Homer N, et al The Sequence Alignment/Map format and SAMtools. Bioinformatics 2009;25:2078–9. 19505943 10.1093/bioinformatics/btp352 PMC2723002

[27] Zhang Y , Liu T, Meyer CA, Eeckhoute J, Johnson DS, Bernstein BE, et al Model-based analysis of ChIP-Seq (MACS). Genome Biol 2008;9:R137. 18798982 10.1186/gb-2008-9-9-r137 PMC2592715

[28] Amemiya HM , Kundaje A, Boyle AP. The ENCODE blacklist: identification of problematic regions of the genome. Sci Rep 2019;9:9354. 31249361 10.1038/s41598-019-45839-z PMC6597582

[29] Ou J , Liu H, Yu J, Kelliher MA, Castilla LH, Lawson ND, et al ATACseqQC: a Bioconductor package for post-alignment quality assessment of ATAC-seq data. BMC Genomics 2018;19:169. 29490630 10.1186/s12864-018-4559-3 PMC5831847

[30] Carroll TS , Liang Z, Salama R, Stark R, de Santiago I. Impact of artifact removal on ChIP quality metrics in ChIP-seq and ChIP-exo data. Front Genet 2014;5:75. 24782889 10.3389/fgene.2014.00075 PMC3989762

[31] Yu G , Wang LG, He QY. ChIPseeker: an R/Bioconductor package for ChIP peak annotation, comparison and visualization. Bioinformatics 2015;31:2382–3. 25765347 10.1093/bioinformatics/btv145

[32] Wu T , Hu E, Xu S, Chen M, Guo P, Dai Z, et al clusterProfiler 4.0: a universal enrichment tool for interpreting omics data. Innovation (Camb) 2021;2:100141. 34557778 10.1016/j.xinn.2021.100141 PMC8454663

[33] Bentsen M , Goymann P, Schultheis H, Klee K, Petrova A, Wiegandt R, et al ATAC-seq footprinting unravels kinetics of transcription factor binding during zygotic genome activation. Nat Commun 2020;11:4267. 32848148 10.1038/s41467-020-18035-1 PMC7449963

[34] Whyte WA , Orlando DA, Hnisz D, Abraham BJ, Lin CY, Kagey MH, et al Master transcription factors and mediator establish super-enhancers at key cell identity genes. Cell 2013;153:307–19. 23582322 10.1016/j.cell.2013.03.035 PMC3653129

[35] Lin CY , Erkek S, Tong Y, Yin L, Federation AJ, Zapatka M, et al Active medulloblastoma enhancers reveal subgroup-specific cellular origins. Nature 2016;530:57–62. 26814967 10.1038/nature16546 PMC5168934

[36] Sherry ST , Ward MH, Kholodov M, Baker J, Phan L, Smigielski EM, et al dbSNP: the NCBI database of genetic variation. Nucleic Acids Res 2001;29:308–11. 11125122 10.1093/nar/29.1.308 PMC29783

[37] Welter D , MacArthur J, Morales J, Burdett T, Hall P, Junkins H, et al The NHGRI GWAS Catalog, a curated resource of SNP-trait associations. Nucleic Acids Res 2014;42:D1001–6. 24316577 10.1093/nar/gkt1229 PMC3965119

[38] Purcell S , Neale B, Todd-Brown K, Thomas L, Ferreira MA, Bender D, et al PLINK: a tool set for whole-genome association and population-based linkage analyses. Am J Hum Genet 2007;81:559–75. 17701901 10.1086/519795 PMC1950838

[39] Gong J , Mei S, Liu C, Xiang Y, Ye Y, Zhang Z, et al PancanQTL: systematic identification of *cis*-eQTLs and *trans*-eQTLs in 33 cancer types. Nucleic Acids Res 2018;46:D971–6. 29036324 10.1093/nar/gkx861 PMC5753226

[40] Carithers LJ , Moore HM. The Genotype-Tissue Expression (GTEx) project. Biopreserv Biobank 2015;13:307–8. 26484569 10.1089/bio.2015.29031.hmm PMC4692118

[41] Li Z , Schulz MH, Look T, Begemann M, Zenke M, Costa IG. Identification of transcription factor binding sites using ATAC-seq. Genome Biol 2019;20:45. 30808370 10.1186/s13059-019-1642-2 PMC6391789

[42] Hnisz D , Abraham BJ, Lee TI, Lau A, Saint-Andre V, Sigova AA, et al Super-enhancers in the control of cell identity and disease. Cell 2013;155:934–47. 24119843 10.1016/j.cell.2013.09.053 PMC3841062

[43] Chen Y , Xu L, Lin RY, Muschen M, Koeffler HP. Core transcriptional regulatory circuitries in cancer. Oncogene 2020;39:6633–46. 32943730 10.1038/s41388-020-01459-w PMC7581508

[44] Saint-Andre V , Federation AJ, Lin CY, Abraham BJ, Reddy J, Lee TI, et al Models of human core transcriptional regulatory circuitries. Genome Res 2016;26:385–96. 26843070 10.1101/gr.197590.115 PMC4772020

[45] Ott CJ , Federation AJ, Schwartz LS, Kasar S, Klitgaard JL, Lenci R, et al Enhancer architecture and essential core regulatory circuitry of chronic lymphocytic leukemia. Cancer Cell 2018;34:982–95. 30503705 10.1016/j.ccell.2018.11.001 PMC6298230

[46] Terekhanova NV , Karpova A, Liang WW, Strzalkowski A, Chen S, Li Y, et al Epigenetic regulation during cancer transitions across 11 tumour types. Nature 2023;623:432–41. 37914932 10.1038/s41586-023-06682-5 PMC10632147

[47] Welsh SJ , Barwick BG, Meermeier EW, Riggs DL, Shi CX, Zhu YX, et al Transcriptional heterogeneity overcomes super-enhancer disrupting drug combinations in multiple myeloma. Blood Cancer Discov 2024;5:34–55. 37767768 10.1158/2643-3230.BCD-23-0062 PMC10772542

[48] Kloet SL , Makowski MM, Baymaz HI, van Voorthuijsen L, Karemaker ID, Santanach A, et al The dynamic interactome and genomic targets of Polycomb complexes during stem-cell differentiation. Nat Struct Mol Biol 2016;23:682–90. 27294783 10.1038/nsmb.3248 PMC4939079

[49] Pique DG , Montagna C, Greally JM, Mar JC. A novel approach to modelling transcriptional heterogeneity identifies the oncogene candidate *CBX2* in invasive breast carcinoma. Br J Cancer 2019;120:746–53. 30820027 10.1038/s41416-019-0387-8 PMC6462018

[50] Vierbuchen T , Ling E, Cowley CJ, Couch CH, Wang X, Harmin DA, et al AP-1 transcription factors and the BAF complex mediate signal-dependent enhancer selection. Mol Cell 2017;68:1067–82. 29272704 10.1016/j.molcel.2017.11.026 PMC5744881

[51] Del Gaudio N , Di Costanzo A, Liu NQ, Conte L, Dell’Aversana C, Bove G, et al CBX2 shapes chromatin accessibility promoting AML via p38 MAPK signaling pathway. Mol Cancer 2022;21:125. 35681235 10.1186/s12943-022-01603-y PMC9178829

[52] Bergeron BP , Diedrich JD, Zhang Y, Barnett KR, Dong Q, Ferguson DC, et al Epigenomic profiling of glucocorticoid responses identifies *cis*-regulatory disruptions impacting steroid resistance in childhood acute lymphoblastic leukemia. Leukemia 2022;36:2374–83. 36028659 10.1038/s41375-022-01685-z PMC9522591

[53] Ma L, , Zou D, , Liu Lin, Shireen H, Abbasi AA, Bateman A, et al Database Commons: a catalog of worldwide biological databases. Genomics Proteomics Bioinformatics 2023;21:1054–8. 36572336 10.1016/j.gpb.2022.12.004 PMC10928426

