# Supplementary material for: HemaCisDB: An Interactive Database for Analyzing Cis-regulatory Elements Across Hematopoietic Malignancies
Source: Genomics Proteomics Bioinformatics. 2024 Dec 26;23(2):qzae088. doi: 10.1093/gpbjnl/qzae088 (PMC12343011; doi:10.1093/gpbjnl/qzae088)
Supplement: qzae088_Supplementary_Data [file qzae088_supplementary_data.zip › supplementary material captions 120924.docx]

**Supplementary material**

**Figure S1 Statistics of H3K27ac ChIP-seq datasets in HemaCisDB**

**A.** Sample sources of H3K27ac ChIP-seq datasets. **B.** Summarization of genetic or drug perturbations across different sample sources for H3K27ac ChIP-seq.

**Figure S2 Data browsing and application modules for H3K27ac ChIP-seq data**

**A.** Data browsing page for H3K27ac ChIP-seq data. **B.** Quality assessment and functional annotation of peaks for selected H3K27ac ChIP-seq dataset. **C.** H3K27ac ChIP-seq peaks identified from selected dataset. Coordinates, fold enrichment, *P* value, *Q* value, and annotation of each peak are reported. **D.** Common SNPs, risk SNPs, and risk SNPs associated with blood disorders that overlap each enhancer region are reported. For each common SNP, corresponding eQTL and LD SNPs in five super-populations (AFR, AMR, EAS, EUR, and SAS) are also reported. **E.** SE identification page. Coordinates, the number of enhancers stitched together, constituent size, signal, rank, overlapped and proximal genes of each SE are reported. SE distribution was plotted with x axis as the rank of SEs and y axis as the level of signals of SEs.

**Table S1 A comprehensive comparison between HemaCisDB and other databases**
