# Supplementary material for: HemaCisDB: An Interactive Database for Analyzing Cis-regulatory Elements Across Hematopoietic Malignancies
Source: Genomics Proteomics Bioinformatics. 2024 Dec 26;23(2):qzae088. doi: 10.1093/gpbjnl/qzae088 (PMC12343011; doi:10.1093/gpbjnl/qzae088)
Supplement: qzae088_Supplementary_Data [file qzae088_supplementary_data.zip › Figure S2 20241209.pdf]

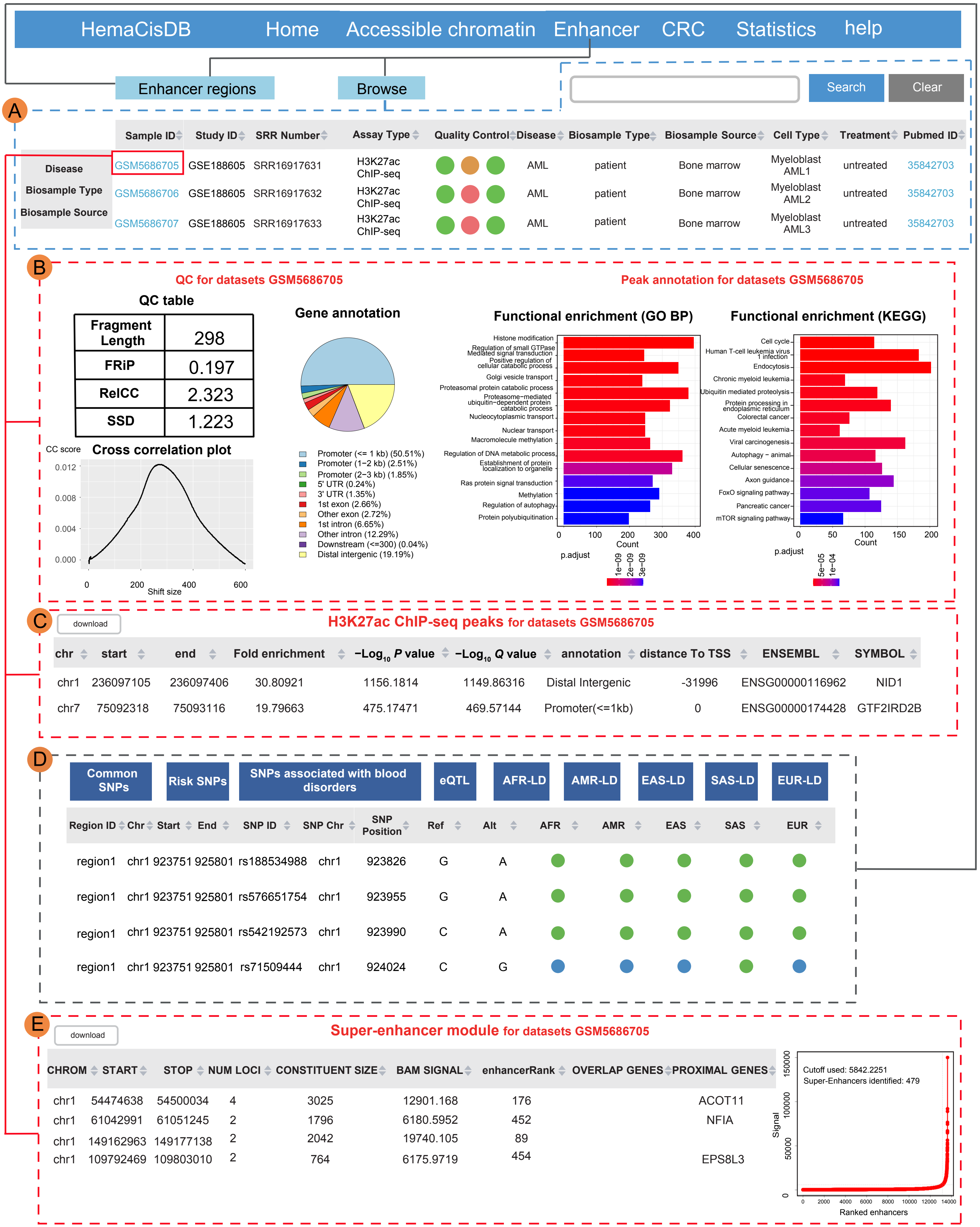

HemaCisDB
Home
Accessible chromatin
Enhancer
CRC
Statistics
help
Enhancer regions
Browse
Search
Clear
A
Sample ID | Study ID | SRR Number | Assay Type | Quality Control | Disease | Biosample Type | Biosample Source | Cell Type | Treatment | Pubmed ID
Disease
Biosample Type
Biosample Source
GSM5686705 GSE188605 SRR16917631 H3K27ac ChIP-seq AML patient Bone marrow Myeloblast AML1 untreated 35842703
GSM5686706 GSE188605 SRR16917632 H3K27ac ChIP-seq AML patient Bone marrow Myeloblast AML2 untreated 35842703
GSM5686707 GSE188605 SRR16917633 H3K27ac ChIP-seq AML patient Bone marrow Myeloblast AML3 untreated 35842703
B
QC for datasets GSM5686705
Peak annotation for datasets GSM5686705
QC table
Fragment Length 298
FRiP 0.197
RelCC 2.323
SSD 1.223
Gene annotation
Promoter (<= 1 kb) (50.51%)
Promoter (1−2 kb) (2.51%)
Promoter (2−3 kb) (1.85%)
5' UTR (0.24%)
3' UTR (1.35%)
1st exon (2.66%)
Other exon (2.72%)
1st intron (6.65%)
Other intron (12.29%)
Downstream (<=300) (0.04%)
Distal intergenic (19.19%)
CC score
Cross correlation plot
0.012
0.008
0.004
0.000
0
200
400
600
Shift size
Functional enrichment (GO BP)
Histone modification
Regulation of small GTPase mediated signal transduction
Positive regulation of cellular catabolic process
Golgi vesicle transport
Proteasomal protein catabolic process
Proteasome−mediated ubiquitin−dependent protein catabolic process
Nucleocytoplasmic transport
Nuclear transport
Macromolecule methylation
Regulation of DNA metabolic process
Establishment of protein localization to organelle
Ras protein signal transduction
Methylation
Regulation of autophagy
Protein polyubiquitination
0 100 200 300 400
Count
p.adjust
1e−09
2e−09
3e−09
Functional enrichment (KEGG)
Cell cycle
Human T-cell leukemia virus 1 infection
Endocytosis
Chronic myeloid leukemia
Ubiquitin mediated proteolysis
Protein processing in endoplasmic reticulum
Colorectal cancer
Acute myeloid leukemia
Viral carcinogenesis
Autophagy − animal
Cellular senescence
Axon guidance
FoxO signaling pathway
Pancreatic cancer
mTOR signaling pathway
0 50 100 150 200
Count
p.adjust
5e−05
1e−04
C
download
H3K27ac ChIP-seq peaks for datasets GSM5686705
chr | start | end | Fold enrichment | −Log10 P value | −Log10 Q value | annotation | distance To TSS | ENSEMBL | SYMBOL
chr1 236097105 236097406 30.80921 1156.1814 1149.86316 Distal Intergenic -31996 ENSG00000116962 NID1
chr7 75092318 75093116 19.79663 475.17471 469.57144 Promoter(<=1kb) 0 ENSG00000174428 GTF2IRD2B
D
Common SNPs
Risk SNPs
SNPs associated with blood disorders
eQTL
AFR-LD
AMR-LD
EAS-LD
SAS-LD
EUR-LD
Region ID | Chr | Start | End | SNP ID | SNP Chr | SNP Position | Ref | Alt | AFR | AMR | EAS | SAS | EUR
region1 chr1 923751 925801 rs188534988 chr1 923826 G A
region1 chr1 923751 925801 rs576651754 chr1 923955 G A
region1 chr1 923751 925801 rs542192573 chr1 923990 C A
region1 chr1 923751 925801 rs71509444 chr1 924024 C G
E
download
Super-enhancer module for datasets GSM5686705
CHROM | START | STOP | NUM LOCI | CONSTITUENT SIZE | BAM SIGNAL | enhancerRank | OVERLAP GENES | PROXIMAL GENES
chr1 54474638 54500034 4 3025 12901.168 176 ACOT11
chr1 61042991 61051245 2 1796 6180.5952 452 NFIA
chr1 149162963 149177138 2 2042 19740.105 89
chr1 109792469 109803010 2 764 6175.9719 454 EPS8L3
Cutoff used: 5842.2251
Super-Enhancers identified: 479
Signal
150000
100000
50000
0
0 2000 4000 6000 8000 10000 12000 14000
Ranked enhancers
