# Supplementary material for: HemaCisDB: An Interactive Database for Analyzing Cis-regulatory Elements Across Hematopoietic Malignancies
Source: Genomics Proteomics Bioinformatics. 2024 Dec 26;23(2):qzae088. doi: 10.1093/gpbjnl/qzae088 (PMC12343011; doi:10.1093/gpbjnl/qzae088)
Supplement: qzae088_Supplementary_Data [file Figure S1 20241209.pdf › Figure S1 20241209.pdf]

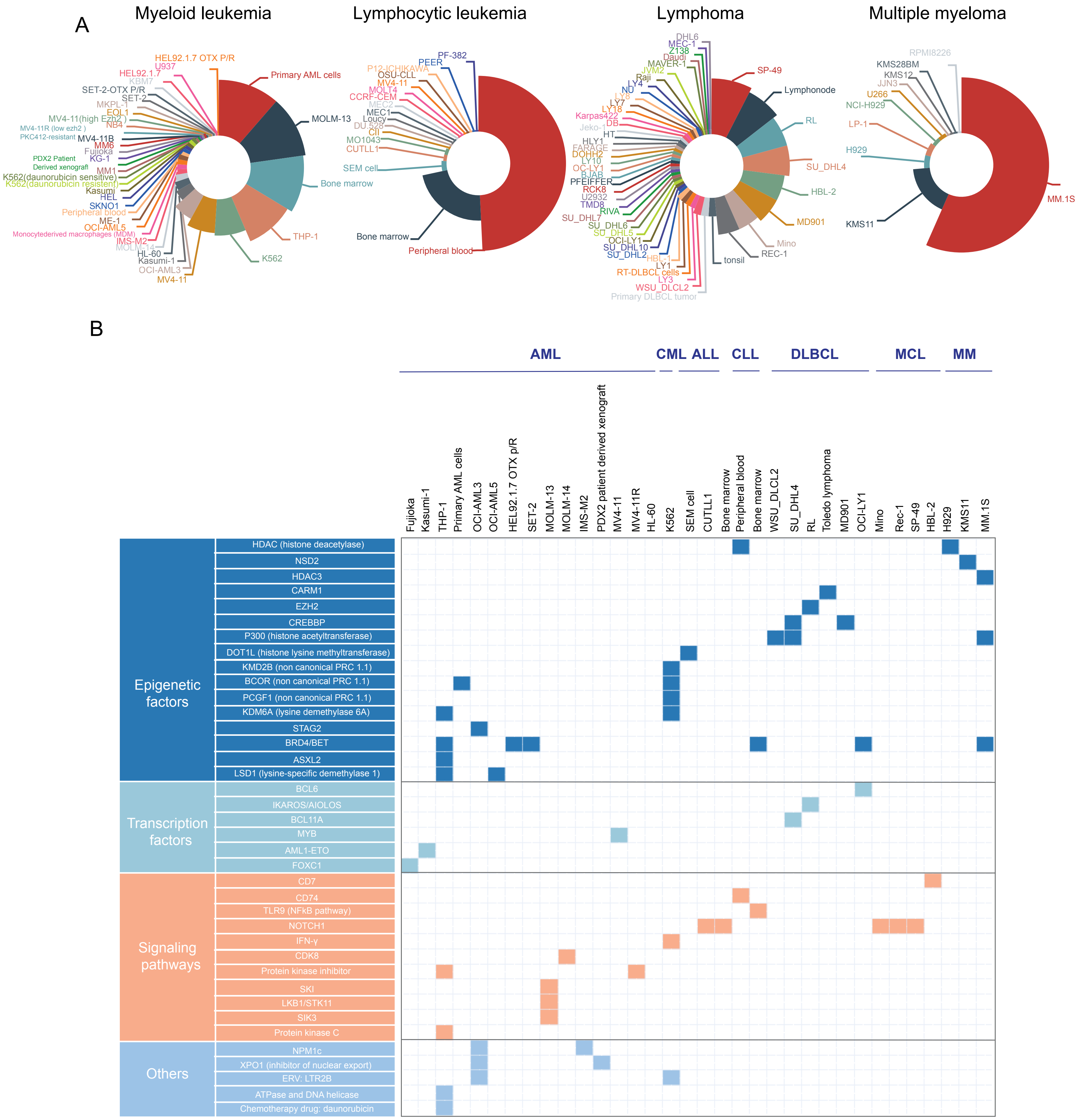
A
Myeloid leukemia
Lymphocytic leukemia
Lymphoma
Multiple myeloma
Primary AML cells
MOLM-13
Bone marrow
THP-1
K562
MV4-11
OCI-AML3
Kasumi-1
HL-60
MOLM-14
IMS-M2
Monocytederived macrophages (MDM)
OCI-AML5
ME-1
Peripheral blood
SKNO1
HEL
Kasumi
K562(daunorubicin resistent)
K562(daunorubicin sensitive)
PDX2 Patient
Derived xenograft
MM1
KG-1
Fujioka
MM6
MV4-11B
PKC412-resistant
MV4-11R (low ezh2 )
MV4-11(high Ezh2 )
NB4
EOL1
MKPL-1
SET-2
SET-2-OTX P/R
KBM7
HEL92.1.7
U937
HEL92.1.7 OTX P/R
PF-382
PEER
P12-ICHIKAWA
OSU-CLL
MV4-11
MOLT4
CCRF-CEM
MEC2
MEC1
Loucy
DU.528
Cll
MO1043
CUTLL1
SEM cell
Bone marrow
Peripheral blood
DHL6
MEC-1
Z138
Daudi
MAVER-1
JVM2
Raji
LY4
NU
LY8
LY7
LY18
Karpas422
DB
Jeko-1
HT
HLY1
FARAGE
DOHH2
LY10
OC-LY1
BJAB
PFEIFFER
RCK8
U2932
TMD8
RIVA
SU_DHL7
SU_DHL6
SU_DHL5
OCI-LY1
SU_DHL10
SU_DHL2
HBL-1
LY1
RT-DLBCL cells
LY3
WSU_DLCL2
Primary DLBCL tumor
SP-49
Lymphonode
RL
SU_DHL4
HBL-2
MD901
Mino
REC-1
tonsil
RPMI8226
KMS28BM
KMS12
JJN3
U266
NCI-H929
LP-1
H929
KMS11
MM.1S
B
AML
CML
ALL
CLL
DLBCL
MCL
MM
Fujioka
Kasumi-1
THP-1
Primary AML cells
OCI-AML3
OCI-AML5
HEL92.1.7 OTX p/R
SET-2
MOLM-13
MOLM-14
IMS-M2
PDX2 patient derived xenograft
MV4-11
MV4-11R
HL-60
K562
SEM cell
CUTLL1
Bone marrow
Peripheral blood
Bone marrow
WSU_DLCL2
SU_DHL4
RL
Toledo lymphoma
MD901
OCI-LY1
Mino
Rec-1
SP-49
HBL-2
H929
KMS11
MM.1S
Epigenetic factors
HDAC (histone deacetylase)
NSD2
HDAC3
CARM1
EZH2
CREBBP
P300 (histone acetyltransferase)
DOT1L (histone lysine methyltransferase)
KMD2B (non canonical PRC 1.1)
BCOR (non canonical PRC 1.1)
PCGF1 (non canonical PRC 1.1)
KDM6A (lysine demethylase 6A)
STAG2
BRD4/BET
ASXL2
LSD1 (lysine-specific demethylase 1)
Transcription factors
BCL6
IKAROS/AIOLOS
BCL11A
MYB
AML1-ETO
FOXC1
Signaling pathways
CD7
CD74
TLR9 (NFkB pathway)
NOTCH1
IFN-γ
CDK8
Protein kinase inhibitor
SKI
LKB1/STK11
SIK3
Protein kinase C
Others
NPM1c
XPO1 (inhibitor of nuclear export)
ERV: LTR2B
ATPase and DNA helicase
Chemotherapy drug: daunorubicin
